# Supplementary figures and images for: Effect of 11-Deoxycorticosterone in the Transcriptomic Response to Stress in Rainbow Trout Skeletal Muscle
Source: Genes (Basel). 2023 Feb 17;14(2):512. doi: 10.3390/genes14020512 (PMC9957386; doi:10.3390/genes14020512)

Principal component scatter plot

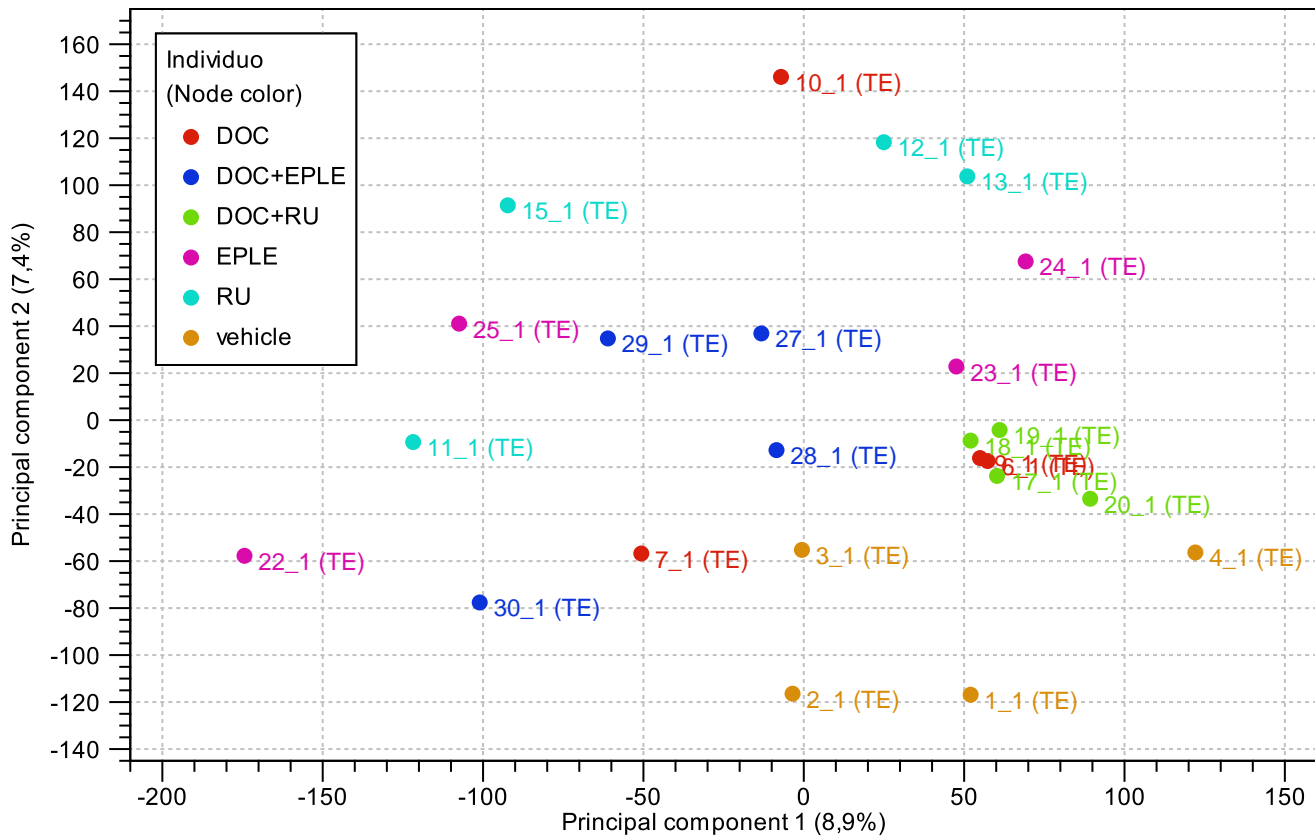

Supplement: Supplementary file 1 [file genes-14-00512-s001.zip › SupplementaryFigureS1.pdf]
